# Update on Fatty Acids and the Brain

**DOI:** 10.3390/nu16244416

**Published:** 2024-12-23

**Authors:** Andrew J. Sinclair

**Affiliations:** 1 Department of Nutrition, Dietetics and Food, School of Clinical Sciences, Monash University, Notting Hill, VIC 3168, Australia; andrew.sinclair@monash.edu; 2 Faculty of Health, Deakin University, Burwood, VIC 3152, Australia

The brain is a lipid-rich organ, mainly due to the very high lipid content of myelin, but in addition to this, all the neuronal cell membranes, of which there are over 80 billion in the human brain [1], contain membrane lipids, typically with a very high proportion of polyunsaturated fatty acids (PUFAs). Docosahexaenoic acid (DHA), which is an *n*-3 PUFA, is the main PUFAs in neuronal cell membranes and, together with arachidonic acid and adrenic acid, makes up the majority of the PUFAs in the brain [1]. Arachidonic and adrenic acids are both *n*-6 PUFAs. These PUFAs, together with palmitic, stearic, and oleic acid; C22:1; C24:0; C24:1; and some long-chain monounsaturated hydroxy fatty acids, are constituents of the phospholipids in the cell membranes of neurons and in myelin [2].

The very high proportion of DHA in the brains of all mammals so far studied (>30) suggests this fatty acid plays major roles in the structure and function of the brain [3]. Evidence gathered from studies on experimental animals has shown that depletion of DHA in the brain and retina, resulting from a deficiency of dietary *n*-3 (omega 3) fatty acids, leads to many subtle changes in brain and retinal function [4]. Interestingly, one behavioural feature of *n*-3 PUFA deficiency in rats is increased aggression [5].

In this series on **Fatty Acids and the Brain**, the authors of one paper (Contribution 1) looked for evidence of DHA deficiency in humans, as judged by increased levels of a tissue fatty acid biomarker (*n*-6 docosapentaenoic acid). The authors concluded that the human populations most likely at risk of *n*-3 deficiency were newborn and weaning infants; infants, children, and adolescents in dryland agriculture areas; and infants and children subject to famines and/or living as refugees. Notably, these populations have rarely been studied.

The high proportion of DHA in the brain has provoked researchers worldwide to examine the effects of DHA supplementation on neural function and look for associations between supplementation with DHA and various neurological conditions. In this series on **Fatty Acids and the Brain**, four papers investigated the effects of DHA supplementation on neural conditions or neural outcomes. Sueyasu et al. (Contribution 2) found that supplementation with a mixture of *n*-3 and *n*-6 PUFAs, combined with lutein and zeaxanthin, did not significantly affect memory function in healthy, non-demented, older individuals with memory complaints, whereas it improved memory function in healthy, non-demented, older individuals with cognitive decline. Iqbal et al. (Contribution 3), in a systematic review involving post-menopausal women, reported that the combined analysis of nine studies did not provide substantial evidence to support the efficacy of *n*-3 PUFAs in improving vasomotor symptoms, sleep quality, and depression. Martinez-Cue et al. (Contribution 4) reviewed the literature on whether fatty acids are a safe tool for improving neurodevelopmental alterations in individuals with Down Syndrome. They reported that the progress recently made in a Down Syndrome mouse model indicated that fatty acids may represent a useful treatment for Down Syndrome. Lecques et al. (Contribution 5) studied the role of endogenously produced *n*-3 PUFAs in the Fat-1 mouse model on neurobehavioral outcomes in mice which had suffered mild traumatic brain injury. Their results demonstrated a protective effect of *n*-3 PUFAs against mild brain injury. Smolińska et al. (Contribution 6) reviewed the impact of fatty-acid-rich diets on central nervous system development and concluded there was a lack of comprehensive synthesis in current research, particularly regarding the broader spectrum of fatty acids and their optimal levels throughout childhood.

Lo et al. (Contribution 7) reviewed the preferred DHA transport species in the brain. They concluded that 1-acetyl,2-docosahexaenoyl-glycerophosphocholine allowed preferential brain uptake of DHA over non-esterified DHA. They suggested that esterification of DHA within DHA-lysophosphatidylcholine or 1-acetyl,2-docosahexaenoyl-glycerophosphocholine might elicit neuroprotective effects against neurological diseases.

The final two papers focus on the detailed compositional aspects of the human brain during ageing and in those with Huntington’s Disease, respectively. Hancock et al. (Contribution 8) concluded that the cerebellum was exceptional in terms of the large number of major phospholipids that underwent changes (with consequential changes in acyl composition) with age, whereas the motor cortex was highly resistant to change. Phillips et al. (Contribution 9) reported that phospholipid alterations in the Huntington’s Disease brain were dependent on the lipid subclass, species, and bond type and the location.

The papers in this series do not delve into the mechanisms of action of the studied PUFAs in the brain, so for some recent information on this topic, readers are referred to an elegant paper by Kim et al. [6], which discusses the neurodevelopmental and neuroprotective actions of DHA. They review the mechanisms involving effects on membranes and metabolite-related signal transduction, with particular emphasis on the roles of DHA-rich phosphatidylserine in these events.

There is much yet to be learned regarding the role of DHA in the brain, as well as that of arachidonic acid, as it is the second most prominent PUFA in neuronal and myelin membranes [7,8]. Both these PUFAs play important roles in structural aspects of neural membranes and signal transduction and as precursors of specialised lipid mediators, such as synaptamide, neuroprostanes, lipoxins, protectins, resolvins, and maresins [9]. The simplified assumption that supplemental DHA (or arachidonic acid) will have functional outcomes in the brain needs to be supported by research delving into possible mechanisms of action. Such research requires the use of cutting-edge tools and techniques and should be conducted in collaboration with nutritionists and those with expertise in neurophysiology [10], biochemistry [11], anatomy [12], and omics techniques, such as connectomics [13].
